# Evaluating Bacillus Calmette–Guérin Polysaccharide Nucleic Acid as an Adjuvant for Influenza Vaccines in Mice

**DOI:** 10.1111/irv.70118

**Published:** 2025-05-07

**Authors:** Sijing Yan, Fan Yang, Jia Ji, Xiantian Lin, Ping Wang, Han Wu, Linfang Cheng, Fumin Liu, Nanping Wu, Hangping Yao, Wade S. J. Wu, Haibo Wu

**Affiliations:** ^1^ State Key Laboratory for Diagnosis and Treatment of Infectious Diseases, National Clinical Research Center for Infectious Diseases, Collaborative Innovation Center for Diagnosis and Treatment of Infectious Diseases, The First Affiliated Hospital, School of Medicine Zhejiang University Hangzhou China; ^2^ Advin Biotech, Inc. San Diego California USA

**Keywords:** adjuvant, BCG polysaccharide nucleic acid, cellular immunity, humoral immunity, influenza vaccine

## Abstract

**Background:**

To enhance influenza vaccine efficacy, it is essential to investigate new adjuvants that are both safe and effective. In a recent study utilizing a mouse model, Bacillus Calmette–Guérin polysaccharide nucleic acid (BCG‐PSN) emerged as a promising candidate vaccine adjuvant.

**Methods:**

This study evaluated the immunomodulatory effects of BCG‐PSN on influenza vaccines hemagglutinin antigen and aluminum adjuvant as controls. Mice were immunized with H1N1 antigen and adjuvants, and serum antibody levels were measured using hemagglutination inhibition, enzyme‐linked immunosorbent assay, and microneutralization assays. Flow cytometry and enzyme‐linked immunospot assays assessed T cell phenotypes and cytokine expression. The protective effects were tested through challenge experiments, and the adjuvants were further evaluated in a quadrivalent seasonal influenza vaccine.

**Results:**

BCG‐PSN adjuvant exhibited favorable safety profiles and demonstrated an ability to elevate total antibody titers, particularly enhancing neutralizing antibody production when co‐administered with the H1N1 antigen. BCG‐PSN increased cytokine levels and the proportion of CD8+ T lymphocytes, indicating its capacity to enhance cellular immunity. Upon viral challenge in mice, BCG‐PSN mitigated the production of inflammatory factors and reduced lung pathology, effectively protecting the mice. Furthermore, BCG‐PSN displayed heightened immunogenicity against a mixture of four antigens.

**Conclusions:**

BCG‐PSN is a reliable and efficient adjuvant for influenza vaccines, holding promise for enhancing vaccine efficacy and increasing immune responses.

## 1 Introduction

Seasonal influenza is a respiratory infection that becomes globally prevalent each year. The incidence of influenza increased dramatically after the phasing out of control measures for coronavirus disease 2019 (COVID‐19) [1]. Influenza A causes pandemics, while influenza B has high morbidity and mortality [2]. Seasonal influenza can result in acute respiratory illness, and in severe cases, it might progress to multi‐organ failure and death [3]. Annually, seasonal influenza is responsible for about 5 million severe cases and 300,000 deaths, accounting for 8.2% of respiratory disease‐related fatalities [4]. Over the past decade, there have been 2.5 influenza‐related outpatient visits per 1000 people across 30 provinces in China [5]. Elevating population antibody levels and controlling the spread of influenza viruses among communities are important to improve public health and alleviate societal burdens.

Vaccination against influenza remains the most cost‐effective way to address this public health problem [6]. The question of how to maximize the immunizing effect with the lowest dose of vaccine has become an urgent issue. Although the immunogenicity of influenza virus antigens allows for the development of adjuvant‐free vaccines against seasonal influenza, certain influenza strains exhibit suboptimal immunogenicity. In the absence of adjuvants, achieving effective protection necessitates either high doses of the vaccine, multiple doses, or a combination of both [7]. Therefore, adjuvants capable of improving antibody responses, increasing efficacy, and reducing the dose of the antigen required to induce protection are essential.

Traditional adjuvants are sometimes associated with pain, fever, and other side effects [8, 9]. Some adjuvants can even produce adverse reactions related to autoimmunity [10]. Previous research indicated that aluminum salts possess certain toxicity, particularly towards the central nervous system [11, 12]. Aluminum in the brain substantially disrupts cellular functions by impeding energy metabolism, phosphorylation, and dephosphorylation processes. It induces modifications in gene expression, diminishes neurotransmitter release, influences ion channel activity, alters membrane properties, and promotes abnormal protein accumulation [13]. The toxicity of aluminum is associated with neurological disorders such as Alzheimer's disease, amyotrophic lateral sclerosis, and autism spectrum disorders (ASD). The incidence of ASD is higher in infants receiving aluminum adjuvants in vaccines [14]. Behavioral changes in mice have been observed following the injection of aluminum adjuvants [15]. Higher aluminum concentrations have been found in the brains of individuals with ASD, suggesting that aluminum adjuvants might be one of the etiological factors of autism. As a vaccine adjuvant, polyinosinic/polycytidylic acid (Poly I:C) demonstrates remarkable efficacy in elevating vaccine antibody levels and augmenting cytotoxic T lymphocyte (CTL)‐dependent cellular immunity [16]. However, its potential to trigger cytokine storms and fever has led to its exclusion from clinical trials [17]. Similarly, the Toll‐like receptor (TLR) 7/8 agonist R848, also effective as a vaccine adjuvant, has raised concerns in the research community because of its toxicity, thus precluding its clinical use [18]. Consequently, there is a widespread consensus within the industry that the development of a novel, efficient adjuvant is urgently required to address the current shortage of vaccine adjuvants.

The Bacillus Calmette–Guérin (BCG) vaccine was originally used to prevent tuberculosis. Phenol extraction of BCG produces BCG polysaccharide nucleic acid (BCG‐PSN), which serves as a nonspecific immunomodulator. It comprises polysaccharides and nucleic acids, including CpG sequences [19]. Approved by the Chinese FDA, this treatment has clinical significance for combatting viral infections, managing allergic conditions, and treating tumors [19, 20, 21, 22]. The phagocytic capacity of alveolar macrophages is substantially boosted by BCG‐PSN [23]. When activated, macrophages can phagocytose intracellular pathogens or promote an immune response by generating nitric oxide and inflammatory cytokines, including tumor necrosis factor alpha (TNF‐α) and interleukin 6 (IL‐6) [24, 25].

This study evaluated the immunomodulatory effects of BCG‐PSN on influenza vaccines using phosphate‐buffered saline (PBS), hemagglutinin (HA) antigen, and aluminum adjuvant as controls. Mice were immunized with H1N1 antigen and adjuvants, and serum antibody levels were measured using hemagglutination inhibition (HAI), enzyme‐linked immunosorbent assay (ELISA), and microneutralization (MN) assays. Flow cytometry (FCM) and enzyme‐linked immunospot (ELISpot) assays assessed T cell phenotypes and cytokine expression. The protective effects were tested through challenge experiments, and the adjuvants were further evaluated in a quadrivalent seasonal influenza vaccine. These findings provide valuable insights for developing novel influenza vaccine adjuvants.

## 2 Materials and Methods

### 2.1 Cells, Proteins, and Viruses

Madin–Darby canine kidney (MDCK) cells were cultured in complete Dulbecco's modified Eagle medium (DMEM, Gibco, Grand Island, New York, United States). The liquid culture medium included 10% fetal bovine serum (FBS, Gibco) and an antibiotic solution (Pen‐Strep, Gibco). The antigens used to immunize mice in this study were all from the HA protein of influenza virus (A/Michigan/45/2015). The virus strain used in the virus challenge experiments was also A/Michigan/45/2015(H1N1). The mAgs included the following four antigens, A/Michigan/45/2015(H1N1), A/Texas/50/2012(H3N2), B/Brisbane/60/2008(V‐like), and B/Phuket/3073/2013(Y‐like). Influenza virus strains and proteins were provided by Zhejiang Tianyuan Biopharmaceutical (Zhejiang, China) and stored at −80 °C.

### 2.2 Adjuvant

Aluminum hydroxide (Al (OH)_3_, Thermo Fisher Scientific, Waltham, Massachusetts, United States) was used as the aluminum adjuvant for this experiment. BCG‐PSN (SiQiKang injection, Hunan SiQi Pharmaceutical Co., Ltd., Hunan, China) was a colorless and clear liquid. The main constituents of BCG‐PSN are polysaccharides and nucleic acids. The polysaccharides, which are present at high levels and are biologically active, are long‐chained, chemically complex macromolecules [19]. A previous study extensively investigated the detailed structure and immunological attributes of BCG‐PSN [25]. These adjuvants were stored at 4 °C.

### 2.3 Animal Experiments

All experiments were conducted utilizing male BALB/c mice aged 6–8 weeks, with six mice assigned to each group. The H1N1 antigen used in this study was administered at a dose of 3 μg per mouse, and for the aluminum adjuvant, the dose was 100 μg per mouse. The animal experiment was approved by the Institutional Animal Care Use Committee (IACUC) of the First Affiliated Hospital of Zhejiang University School of Medicine (No. 2020–181).

### 2.4 Enzyme‐Linked Immunosorbent Assay (ELISA)

The H1N1 HA protein (A/Michigan/45/2015) was added to a 96‐well enzyme labeling plate at 20 ng per well, along with the coating solution. The plate was then incubated at 4 °C for 16 h. After washing, the plates were blocked for 2 h at room temperature. Then, 5 μL of undetected serum samples were diluted 1000‐fold with PBS at a 2‐fold ratio, added to the wells, and incubated for 1 h at 37 °C. After washing, the plates were incubated with horseradish peroxidase (HRP)‐conjugated goat anti‐mouse IgG (Novus Biologicals, Englewood, Colorado, United States) for 30 min at 37 °C. In addition, IgG typing was performed using HRP‐labeled goat anti‐mouse IgG1, IgG2a, IgG2b, and IgG3 secondary antibodies (Southern Biotech, Birmingham, Alabama, United States). After washing, 3,3′,5,5′‐tetramethylaniline (TMB) was added and incubated in the dark to produce a colorimetric reaction. Finally, the reaction was stopped by adding termination solution and the optical density (OD) was read at 450 nm [26]. The endpoint dilution for each sample was determined as 2.1 times the OD value of the PBS group.

### 2.5 Hemagglutination Inhibition Assay (HAI)

An HAI assay was employed to determine the hemagglutination titer [27]. The virus was diluted to four hemagglutination units (HAU)/25 μL. A receptor‐disrupting enzyme was added for inactivation, which was incubated at 56 °C for 30 min. A 2‐fold multiplicative dilution was performed using a 10 μg/mL dilution of the serum to be tested as the starting concentration. Next, 4 HAU of the virus was added and incubated for 30 min at room temperature. The HI titer was titrated using 1% chicken red blood cells, and was defined as the lowest concentration at which no agglutination was observed.

### 2.6 Micro‐Neutralization Assay (MN)

MDCK cells inoculated with the viral solution were incubated in a CO_2_ incubator until the appearance of cytopathic effects (CPE) was observed. The concentration of viral solution that caused 50% of the cells to develop CPE was determined as the half tissue culture infection dose (TCID50) [28]. One day in advance, 2 × 10^4^ MDCK cells were inoculated into one well of a 96‐well plate. A blank 96‐well plate was taken and the serum was diluted 2‐fold with 10 μg/mL of viral diluent. Additional negative and positive cell control wells were established. The serum was mixed with an equal volume of 100 TCID50 virus and incubated in a 5% CO_2_ incubator at 37 °C for 2 h. The entire mixture of virus and serum was added to MDCK cells in plates. The virus diluent was prepared by adding 6.6 mL of 7.5% BSA, 5 mL of double antibiotics, and 500 μg of TPCK to DMEM, and adjusting the final volume to 50 mL. Virus diluent was added to the negative control wells and 100 TCID50 of virus was added to the positive control wells. The entire plate was incubated at 37 °C and 5% CO_2_ for 72 h, and the results were analyzed by detecting the level of CPE [29].

### 2.7 Splenic Cell Extraction

The acquisition of splenic cells was conducted using a mouse spleen tissue dissociation kit from Miltenyi Biotec (Bergisch Gladbach, Germany). Following the manufacturer's guidelines, the mouse splenocytes were dissociated and subsequently stored at −80 °C.

### 2.8 Enzyme‐Linked Immunospot Assay (ELISpot)

The ELISpot assay utilized a commercial kit ELISpot Plus (Mabtech, Stockholm, Sweden). Following the supplier's instructions, mouse splenic cells were processed, and the resulting spots were observed and counted under a fluorescence enzyme‐linked immunospot analyzer or a dissecting microscope.

### 2.9 Flow Cytometry (FCM) Analysis

The FCM analysis was conducted based on cues from prior research [30]. Lymphocytes were suspended in 1 mL of Roswell Park Memorial Institute (RPMI) 1640 supplemented with 10% FBS and transferred to a 24‐well plate for counting and documentation. The cells were diluted to a concentration of 2 × 10^6^/mL for testing. After collection, cells from wells were centrifuged at 300 × *g* for 5 min to remove the supernatant. Each well was then treated with 200 μL of PBS, centrifuged again at 300 × *g* for 5 min, and approximately 50 μL of liquid was left in the well. The bottom sediment was dispersed using a microplate shaker. Subsequently, 250 μL of cell viability dye (BD Biosciences, San Jose, California, United States; 1000‐fold dilution) was added to each well. After centrifugation at 300 × *g* for 5 min and removal of the supernatant, approximately 50 μL of liquid was left in each well. The bottom sediment was dispersed using a microplate shaker. CD45, CD3, CD4, CD8, CD62L, and CD44 antibodies (BD Biosciences) were diluted 1:1000 in PBS containing 2% FBS, and 80 μL of the diluted solution was added to each well for incubation at room temperature in the dark for 20 min. Following centrifugation at 300 × *g* for 5 min and removal of the supernatant, 150 μL of cell fixation solution (BD Biosciences) was added to each well and incubated at room temperature in the dark for 30 min. Finally, the cells were centrifuged at 450 × *g* for 5 min, the supernatant was removed, and the cells were resuspended in 400 μL of PBS containing 2% FBS before being analyzed using a flow cytometer.

### 2.10 Cytokine Assays

The concentrations of cytokines IFN‐γ, IL‐1ß, IL‐2, IL‐4, IL‐5, IL‐6, IL‐10, Keratinocyte chemoattractant (KC)/human growth‐regulated oncogene (GRO), and TNF‐α in the supernatant of mouse splenocytes were assessed using the MSD V‐Plex Proinflammatory Panel 1 Mouse Kit (Meso Scale Diagnostics, Rockville, Maryland, United States). A standard curve was constructed to determine suitable dilutions for analysis. Accordingly, splenocyte supernatant samples were diluted at a 1:2 ratio. The MSD procedure was conducted following the manufacturer's instructions.

Commercial ELISA kits (4A Biotech, Beijing, China) were used for the cytokine assay post‐viral challenge. Mouse sera were processed following the manufacturer's guidelines. The OD at 450 nm was then measured using a microplate reader.

### 2.11 Histopathology

Mouse lung tissues were preserved by fixation in formalin. Referring to a previous study [31], the tissues were stained with hematoxylin and eosin (HE). Indicators, such as the thickness of neutrophils and neutrophils in the alveoli and the alveolar interstitium, were observed under a light microscope. The lungs from age‐matched mice in the PBS and aluminum adjuvant groups served as negative and positive controls, respectively.

### 2.12 Statistical Analysis

Statistical analysis was conducted using Graph Pad Prism software (GraphPad Inc., La Jolla, California, United States). *p* Values were determined using one‐way analysis of variance (ANOVA). Significance levels were denoted as follows: *p < 0.05 indicates statistically significant, **p < 0.01 indicates highly statistically significant, and ***p < 0.001 indicates extremely statistically significant.

## 3 Results

### 3.1 Safety Analysis of BCG‐PSN as an Adjuvant

**FIGURE 1:**
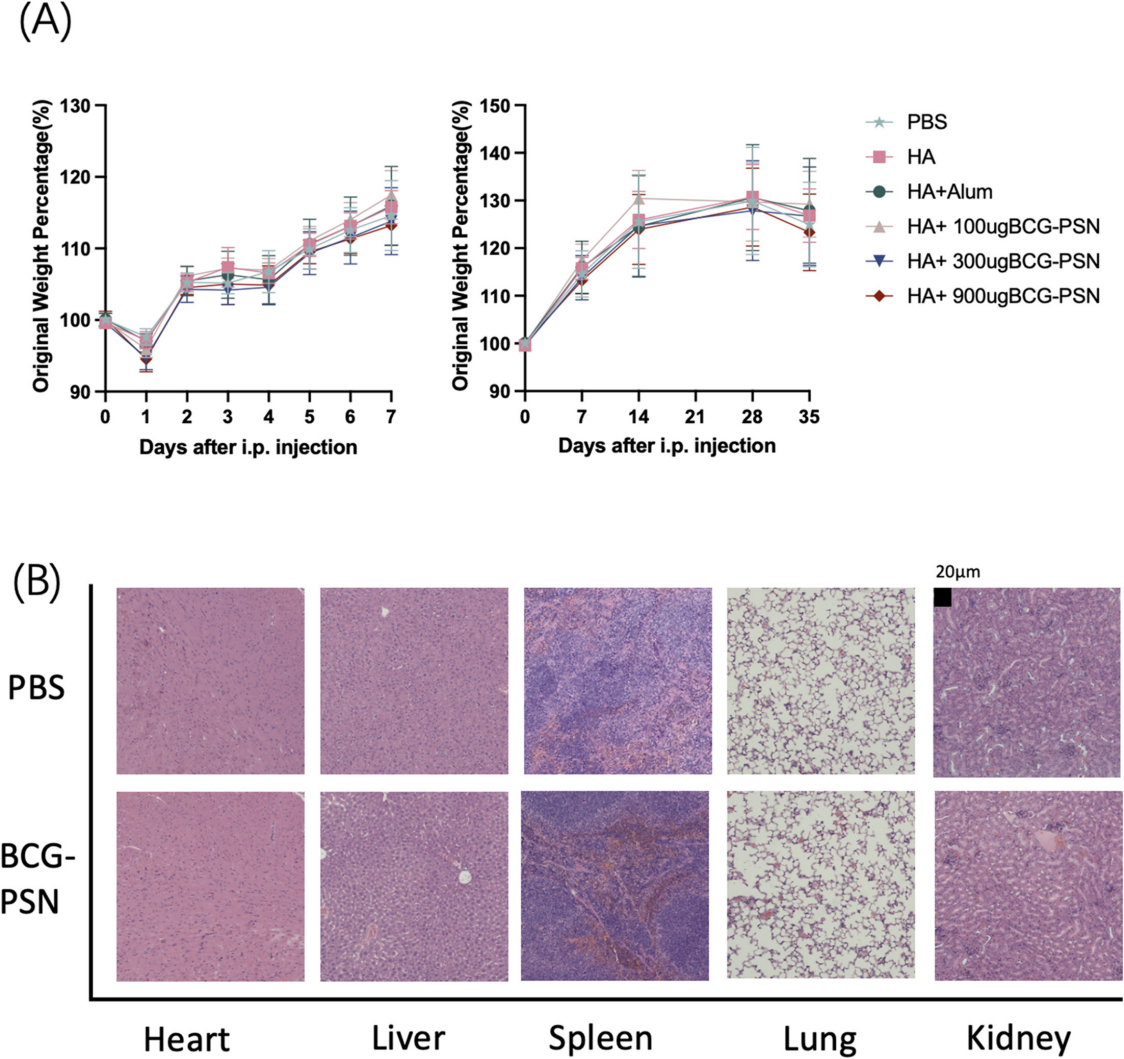
Safety analysis of the adjuvant. (A) Evaluation of mouse body weight changes relative to their initial weight after primary immunization. (B) Histological assessment of major organs (including the heart, liver, spleen, lungs, and kidneys) from mice treated with a high dose of BCG‐PSN (900 μg) following hematoxylin and eosin (H&E) staining.

To investigate the safety of the adjuvant, this study assessed the body weight of mice after the initial immunization. Additionally, organs such as the heart, liver, spleen, lungs, and kidneys were collected and subjected to HE staining to observe pathological changes under an optical microscope. As depicted in Figure 1A, there were no statistically significant variances in the percentage change in body weight across any of the groups at each time point, aligning with the expected pattern of normal mouse growth. Moreover, as illustrated in Figure 1B, no anomalies were noted in the visual appearance of mouse organs during postmortem sampling, and there were no evident pathological alterations observed in HE‐stained tissue sections. Throughout the experiment, no abnormal signs were observed in the mice, indicating that BCG‐PSN has a favorable safety profile.

### 3.2 Dose‐Dependent Effects of the BCG‐PSN Adjuvant

**FIGURE 2:**
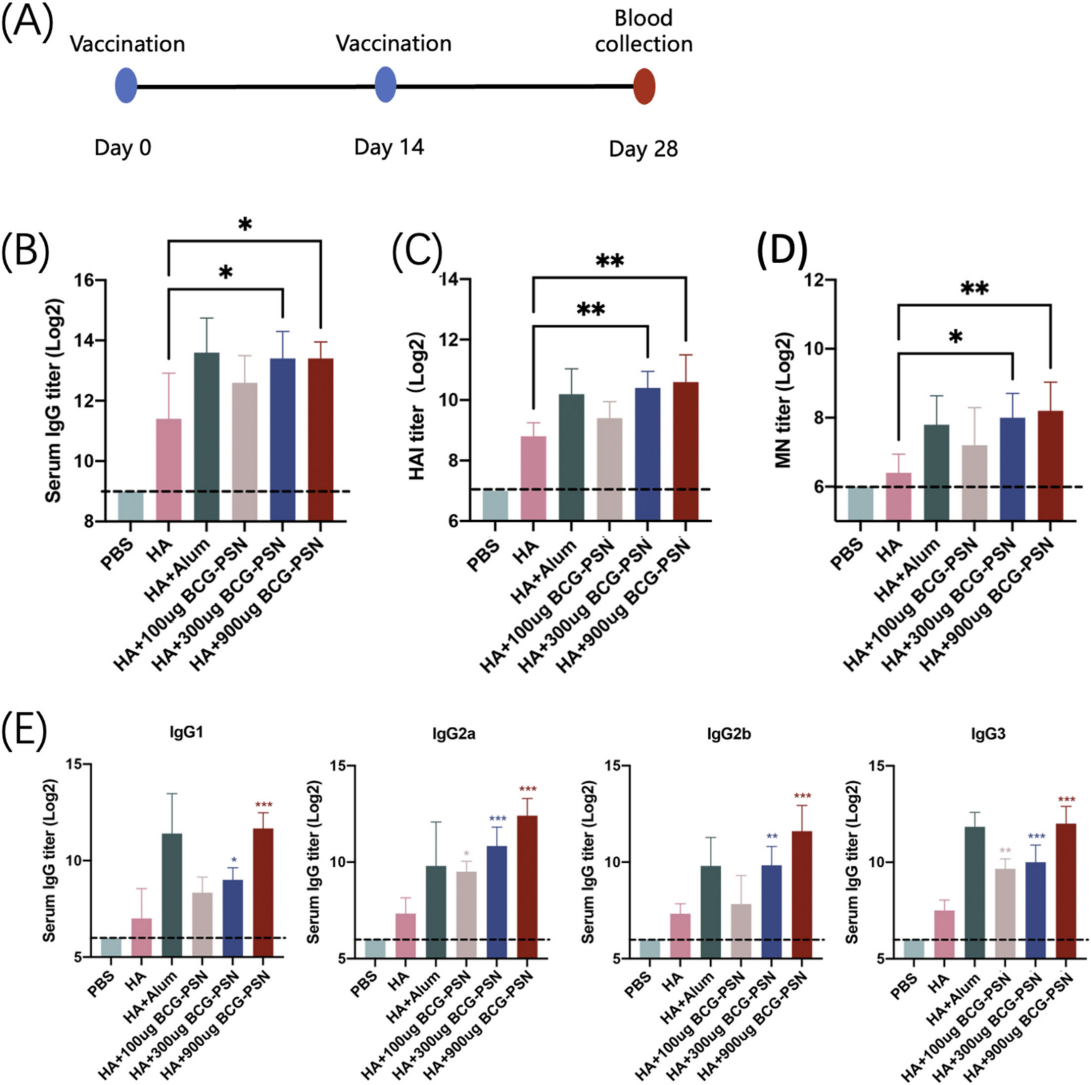
Dose‐dependence of BCG‐PSN. (A) Vaccination, blood collection, and challenge schedule. (B) IgG titers produced by mice against antigens and adjuvants. The limit of detection was 512 (indicated by the dashed line). (C) Hemagglutination inhibition of antibodies produced by mice against antigens and adjuvants. The limit of detection was 256 (indicated by the dashed line). (D) Neutralization of antibodies produced by mice against antigens and adjuvants. The limit of detection was 128 (indicated by the dashed line). (E) Titers of the four IgG isotypes produced by mice against antigens and adjuvants. The limit of detection was 128 (indicated by the dashed line). *p* Values were calculated using one‐way ANOVA. *p < 0.05, **p < 0.01, and ***p < 0.001.

Three different doses of BCG‐PSN (100, 300, and 900 μg) were mixed with the H1N1 antigen for immunization. The H1N1 antigen (3 μg) together with adjuvant or PBS were administered to the mice by intraperitoneal injection [32]. A second immunization was given 2 weeks after the first immunization. Retro‐orbital venous blood collection under anesthesia was performed on the mice on day 28 after the first immunization (Figure 2A). The results showed that the IgG titers in the BCG‐PSN group were 4‐fold higher than those in the HA group at adjuvant doses of 300 and 900 μg (Figure 2B). Additionally, the BCG‐PSN adjuvant induced a more robust hemagglutination inhibition effect, exhibiting a significant difference compared with that of the non‐adjuvant group (Figure 2C). Similar trends were observed in the results of the MN assays (Figure 2D). IgG1, IgG2a, IgG2b, and IgG3 titers were largely consistent with the levels of total IgG antibodies (Figure 2E). Thus, BCG‐PSN enhanced the immune response and is dose‐dependent in the range of 100 to 900 μg.

### 3.3 Impact of the BCG‐PSN Adjuvant on Cellular Immunity in Immunized Mice

**FIGURE 3:**
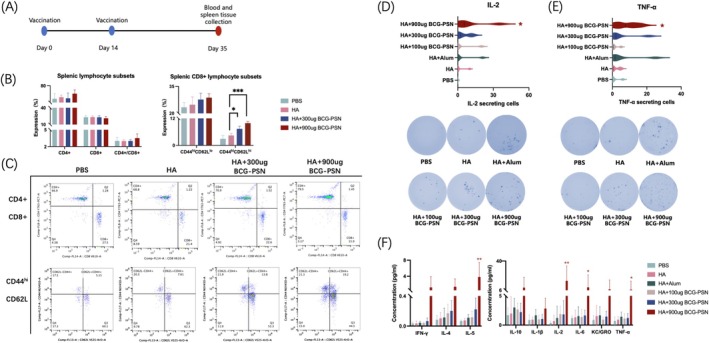
The impact of BCG‐PSN adjuvant on mouse cell immunity. Splenocytes were collected for flow cytometry analysis in the third week after booster immunization. In the third week after booster immunization, splenocytes were collected for ELISpot assays and cytokine detection. (A) Vaccination, blood and spleen tissue collection schedule. (B) Statistical data on the proportions of T lymphocyte subsets in splenocytes. (C) Representative flow cytometry analysis plots for each experimental group. (D,E) Number of spots produced by splenocyte supernatants targeting cytokines IL‐2 and TNF‐α. (F) Concentration of various cytokines in mouse splenocyte supernatants, as determined using a commercial MSD assay kit. *p* Values were calculated using one‐way ANOVA. *p < 0.05 and**p < 0.01.

In the third week post‐secondary immunization (Figure 3A), splenocytes from the mice were harvested for FCM analysis. Surface staining of spleen cells was conducted to investigate the impact of each adjuvant on the ratio of T lymphocyte subsets and to compare the effects of the adjuvants on the differentiation of memory cells (Figure 3B,C). The results revealed that mice co‐immunized with BCG‐PSN adjuvant and H1N1 influenza virus antigen exhibited increased numbers of CD4+ and CD8+ T lymphocytes. Immunization with BCG‐PSN at a dose of 900 μg elevated the proportion of CD4+ T lymphocytes and the CD4+/CD8+ ratio. Although there was no significant alteration in the proportion of CD8+ T lymphocytes, there were diverse increases in the proportions of central memory cells (CD44^hi^ CD62L^hi^) and effector memory cells (CD44^hi^ CD62L^lo^) within the CD8+ T lymphocyte population. Particularly notable was the significant increase in the proportion of central memory cells within the CD8+ T lymphocytes, rising from 4 to 9%.

The secretion of cytokines in the supernatant of splenocyte cultures was evaluated using the ELISpot assay to assess the adjuvant's effect on cellular immunity in mice. The spot count in the BCG‐PSN adjuvant group surpassed that in the HA group, reaching its peak at a BCG‐PSN dose of 900 μg (Figure 3D,E). Similar results were obtained when the samples were tested using a multiplex assay kit. Significant differences in the concentrations of IL‐2, IL‐5, IFN‐γ, and TNF‐α were observed between the high‐dose (900 μg) BCG‐PSN group and the HA group (Figure 3F). This indicated that mice co‐immunized with BCG‐PSN and H1N1 influenza virus antigen exhibited enhanced cellular immunity through the promotion of T lymphocyte proliferation, differentiation, and cytokine secretion in response to H1N1 influenza virus antigen stimulation in vitro.

### 3.4 Improvement in the Survival Rate and Infection Resistance of Mice Caused by the BCG‐PSN Adjuvant

**FIGURE 4:**
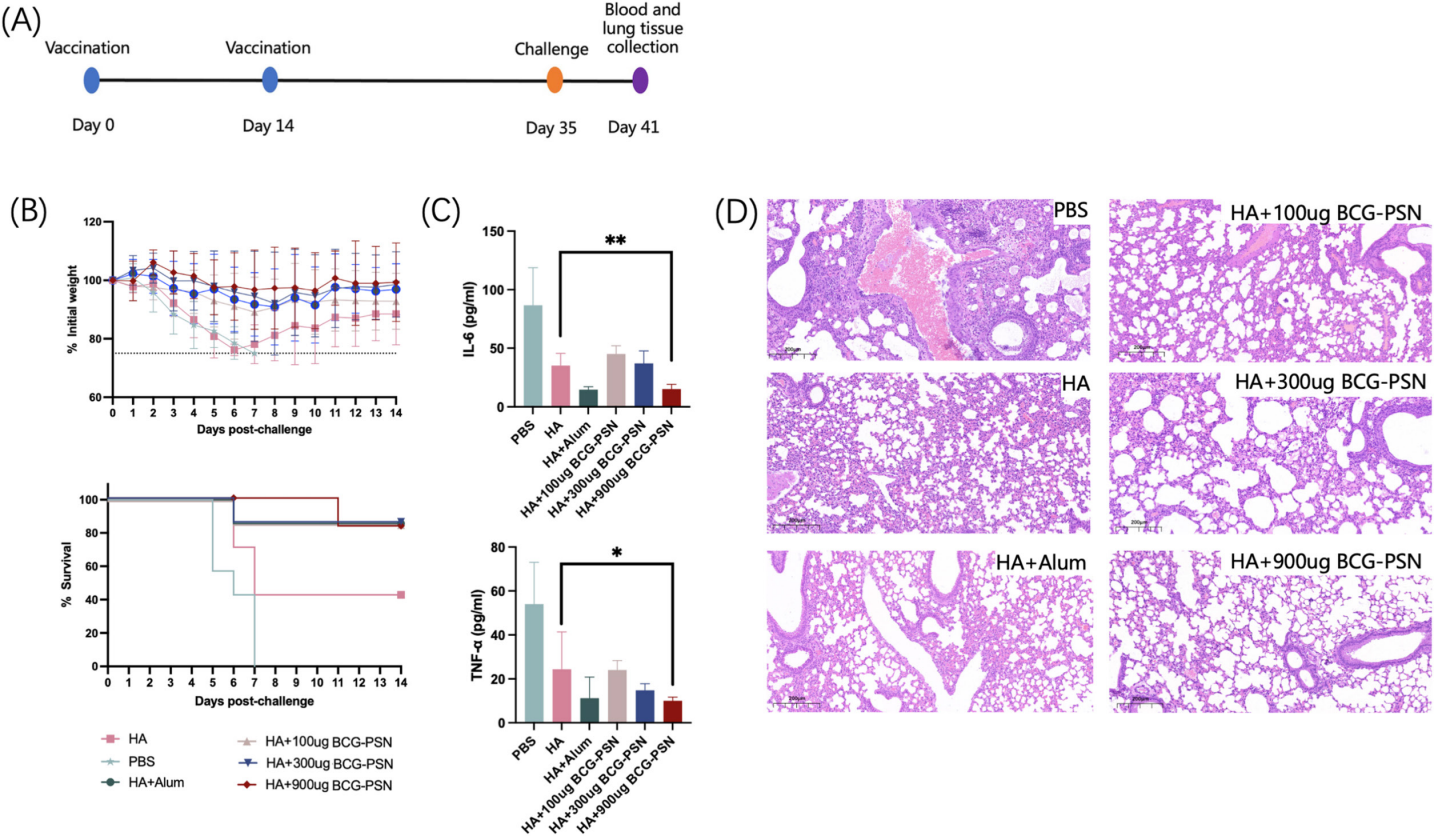
Anti‐infective effects of the BCG‐PSN adjuvant in mice. (A) Vaccination, challenge, blood, and lung tissue extraction schedule. (B) Changes in body weight of mice subjected to viral challenge (above) and survival curve (below). (C) Serum levels of cytokines IL‐6 and TNF‐α in mice at 6 days after receiving the challenge. (D) Representative image of lung tissue under a light microscope from mice after virus challenge. *P*‐values were calculated using one‐way ANOVA. *p < 0.05 and**p < 0.01.

To analyze the anti‐infective effect of BCG‐PSN, a virus challenge test (Figure 4A) was performed by intranasal inoculation with five 50% mouse lethal dose (MLD50). Vaccine efficacy in the HA group was 60%, which increased to 80% when combined with the BCG‐PSN adjuvant. On day 6 after the first intraperitoneal injection of 3 μg of H1N1 antigen, mice were challenged with virus and body weight changes were recorded. A 25% loss in body weight of the mice was used as the humane endpoint. Mice immunized with BCG‐PSN lost less than 10% of their body weight and had no significant trough. More than 80% of mice in the BCG‐PSN group survived the 14‐day viral challenge. In contrast, in the non‐adjuvant group, their body weight decreased to 75% of the original body weight around day 6 and showed a mortality rate of more than 60%. Therefore, mice in the adjuvant group had an increased survival rate and a smaller change in body weight (Figure 4B). This indicated that the adjuvant has a protective effect against influenza virus infection in mice.

Subsequently, lung tissue was taken from mice at 6 days after viral challenge and pathomorphological observations were performed as described previously [31]. Cytokine levels, including IL‐6 and TNF‐α, in the lungs of mice were also assessed. The results showed that the BCG‐PSN adjuvant also had a beneficial effect on reducing the inflammatory response (Figure 4C). The non‐adjuvant group showed significant inflammatory cell aggregation around the alveoli and bronchi, while the adjuvant group showed significantly reduced inflammation (Figure 4D). At a BCG‐PSN dose of 900 μg, the levels of inflammatory factors were notably diminished, reaching their nadir and demonstrating a significant reduction compared that in the non‐adjuvant group. Despite some individual variance being observed in the viral challenge test, it was evident that the BCG‐PSN adjuvant could enhance the mice's resistance to infection.

### 3.5 Performance of the BCG‐PSN Adjuvant in a Quadrivalent Mixed Antigen

**FIGURE 5:**
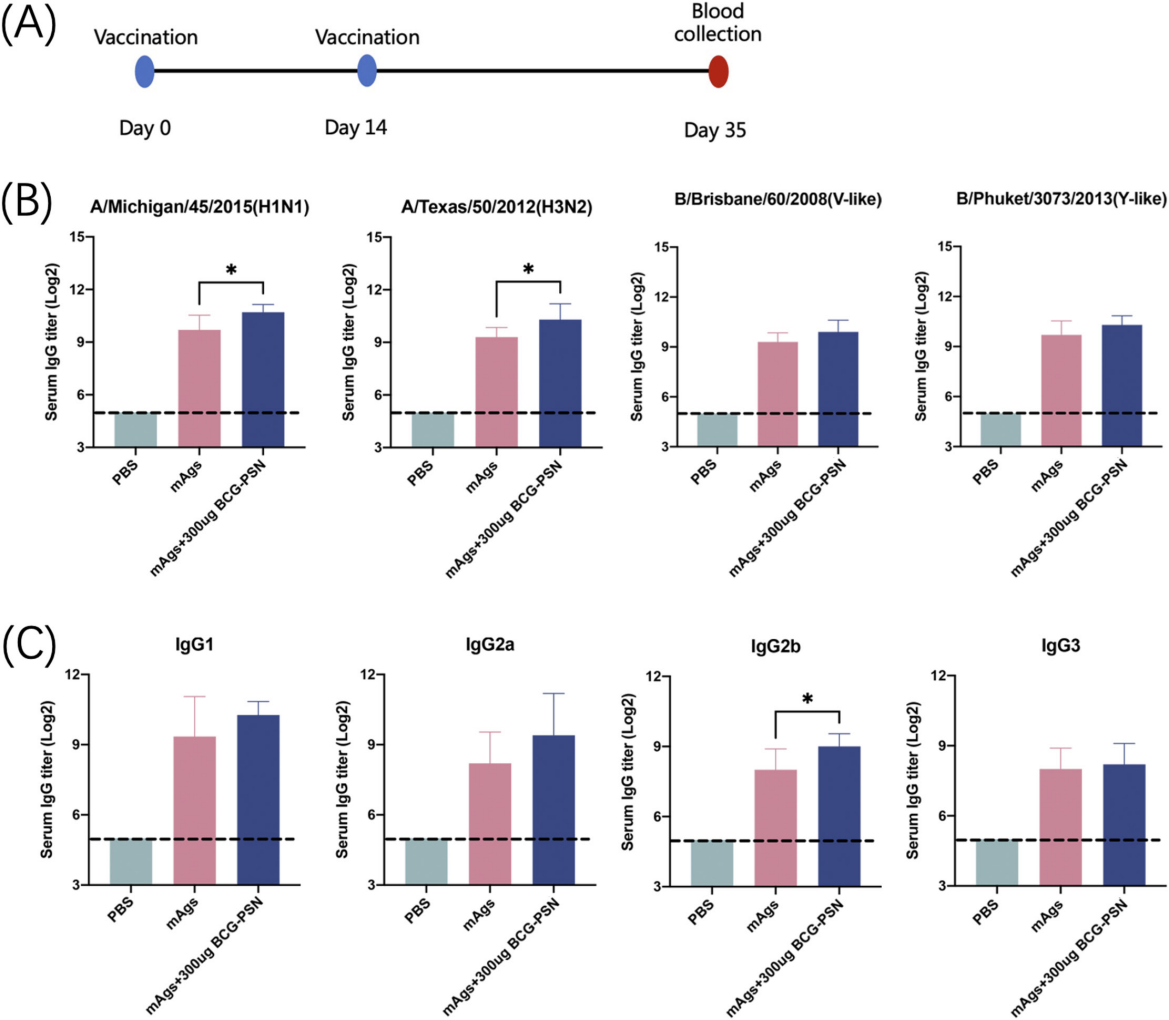
Adjuvant effect of BCG‐PSN in four mixed antigens (mAgs). (A) Vaccination and blood collection schedule. (B) Specific serum IgG titers produced by mice against the four different antigens. The limit of detection was 64 (indicated by the dashed line). (C) Titers of IgG1, IgG2a, IgG2b, and IgG3 produced by mice against the H1N1 antigen when co‐immunized with mAgs and adjuvants. The limit of detection was 64 (indicated by the dashed line). *p* Values were calculated using one‐way ANOVA. *p < 0.05 and**p < 0.01.

We also explored the role of the BCG‐PSN adjuvant in the presence of four mixed antigens (mAgs). The mAgs included the most popular vaccine strains, including A/Michigan/45/2015(H1N1), A/Texas/50/2012(H3N2), B/Brisbane/60/2008(V‐like), and B/Phuket/3073/2013(Y‐like). The dose of each antigen in the mAg was 1.5 μg. Immunizations and blood collection were performed as described previously (Figure 5A). We performed ELISA on sera from mice at 35 days after the initial immunization. We found that the total IgG titers against influenza A antigens (H1N1 and H3N2) in the BCG‐PSN group were twice as high as those in the non‐adjuvanted group. For the four mAgs, the BCG‐PSN adjuvant promoted four subtypes of IgG1, IgG2a, IgG2b, and IgG3 to varying degrees. Therefore, as an adjuvant, BCG‐PSN also displayed an immune enhancing effect on the four mAgs (Figure 5B,C).

## 4 Discussion

While current influenza vaccines demonstrate some immunogenicity, the levels of antibodies generated within the population remain relatively low, failing to achieve the desired efficacy. Thus, influenza remains a substantial threat to public health. Adjuvants, comprising substances added to vaccines to enhance immune responses to antigens, play roles in improving antigen immunogenicity, accelerating vaccine responses, promoting immune regulation, and enhancing vaccine stability [33, 34, 35]. In influenza vaccines, aluminum adjuvants are the most commonly used adjuvants [36]. In addition to aluminum adjuvants, various other adjuvants, such as MF59 and AS03, are employed for the clinical administration of influenza vaccines [37, 38]. Nevertheless, traditional adjuvants come with limitations, including inducing fever and headache, disrupting cell metabolism, and potentially triggering autoimmune diseases [10, 11, 12]. Therefore, it is crucial to develop novel adjuvants that are both safe and effective.

As an immunomodulator, BCG‐PSN has been extensively applied in clinical environments. For instance, the administration of BCG‐PSN can trigger a Th1‐mediated immune response. This occurs by enhancing T cell differentiation into the Th1 subset and the activation of the TLR signaling pathway. Consequently, Th1‐type cytokines, such as IL‐2 and IFN‐γ, are secreted [22]. In addition, BCG‐PSN has shown potential in patients with chronic urticaria. It fosters IL‐2 production via the Th1 pathway while suppressing IL‐10 production through the Th2 pathway. Additionally, it diminishes β‐hexosaminidase release and modulates mast cell activation mediated by IgE [39]. In individuals with pulmonary tuberculosis, BCG‐PSN demonstrated a notable capability to augment the phagocytic ability of alveolar macrophages. These macrophages play a pivotal role in the innate immune system and participate in adaptive immune reactions. Upon stimulation, they promote immune responses by producing nitric oxide (NO) and inflammatory cytokines, such as TNF‐α, IFN‐γ, IL‐10, and IL‐6 [40].

**FIGURE 6:**
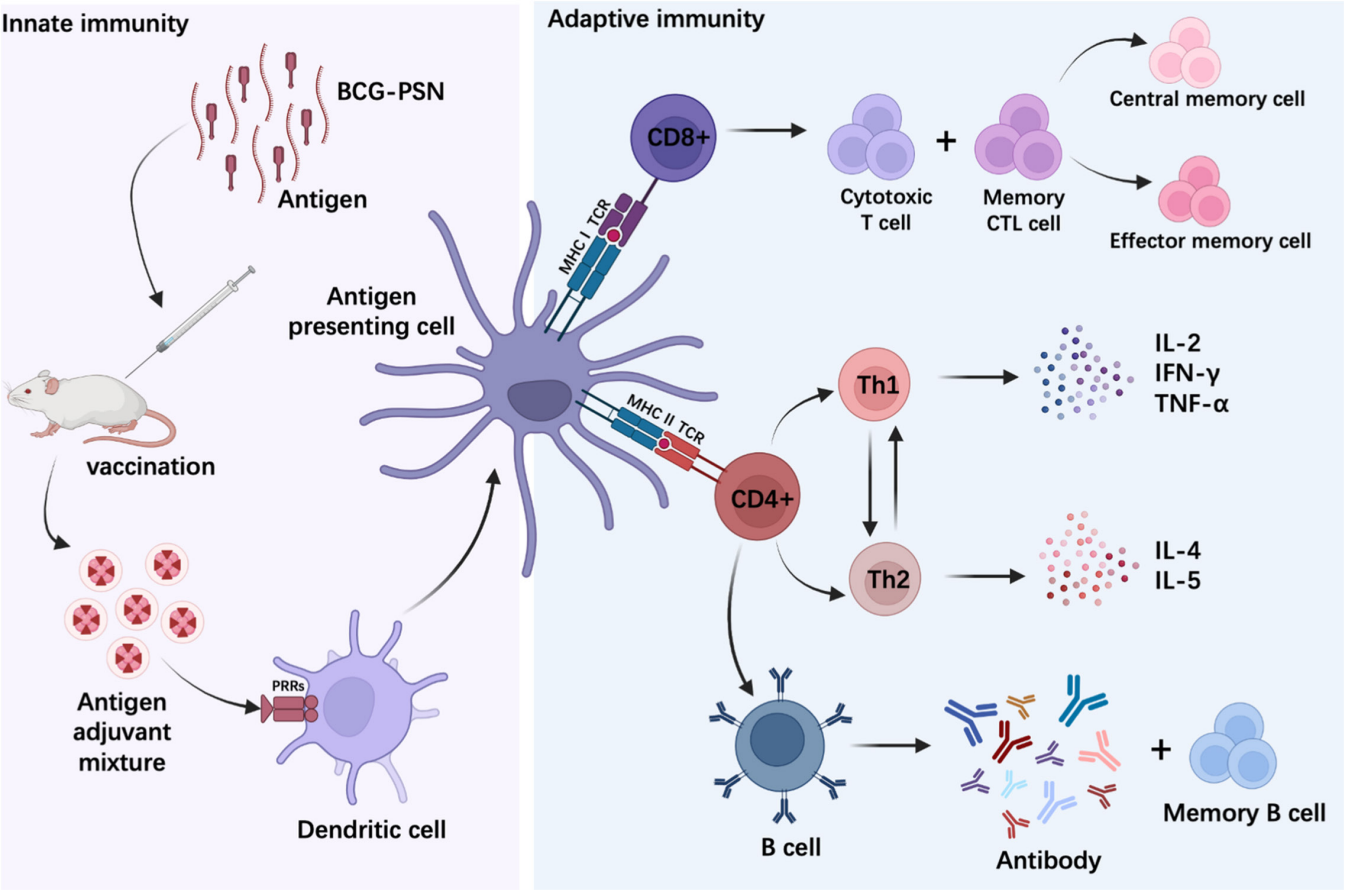
Schematic diagram depicting the enhanced immune response mechanism in hosts following co‐immunization with BCG‐PSN adjuvant and influenza vaccines. The image was created using BioRender software (https://biorender.com).

As an adjuvant for influenza vaccines, BCG‐PSN follows a fixed mechanism of action. It can be recognized by pattern recognition receptors (PRRs), thereby triggering innate immunity [41]. This crucial immunomodulator activates immature dendritic cells (DCs) to become antigen‐presenting cells (APCs) [42]. Subsequently, MHC I and MHC II on the surface of APCs bind to immature CD8+ and CD4+ lymphocytes, inducing the proliferation and differentiation of T lymphocytes, leading to adaptive immunity [43]. The main mechanism of action of BCG‐PSN is summarized in Figure 6, based on the results of this study. When co‐immunized with DNA vaccines, BCG‐PSN was found to significantly reduce the required dosage of DNA vaccines. Co‐immunization with 10 μg of DNA vaccine and BCG‐PSN triggered robust cellular and humoral immune responses, mirroring those induced by the sole administration of 100 μg of the DNA vaccine [22]. Furthermore, it was observed that co‐immunization with BCG‐PSN enhanced the antibody titer against an infectious bursal disease virus vaccine in poultry. Additionally, BCG‐PSN adjuvant led to an increase in the CD4+/CD8+ ratio and the relative thymus weight [44].

This investigation focused on assessing BCG‐PSN's potential as an adjuvant for influenza vaccines. Whether co‐immunized with a single antigen or used as a vaccine adjuvant in multi‐antigen formulations, it significantly increased the total IgG and various IgG subtype antibody titers, and promoted the production of neutralizing antibodies. The IgG2a/IgG2b ratio in the BCG‐PSN adjuvant group was two to four times higher than that in the group without the adjuvant, with the most significant enhancement observed at 100 μg of BCG‐PSN. Furthermore, in the quadrivalent mixed antigen test, the IgG2a/IgG2b ratio was also increased compared with that in the control group. This indicated that BCG‐PSN, whether in single antigen or multi‐valent vaccines, contributes to the induction of Th1‐type immune responses. In vaccine immunization, Th1/Th2 responses are pivotal, serving as primary regulatory focuses for vaccine adjuvants [45]. Emulsion adjuvants, such as MF59 and AS03, are capable of eliciting combined Th1/Th2 T‐cell responses [46]. However, the commonly employed aluminum adjuvant cannot promote Th1 cell‐mediated immunity [47]. The BCG‐PSN adjuvant can effectively complement this deficiency.

Cytokines play a critical role in Th immune responses [48]. For example, IL‐12 facilitates Th1 cell differentiation, leading to the production of IFN‐γ, TNF‐α, and IL‐2. Additionally, it activates CD8+ CTLs, thus fostering B cell synthesis of IgG and orchestrating cellular immunity [49]. The cellular immunomodulatory effects of the BCG‐PSN adjuvant were analyzed through ELIspot and FCM. Splenocytes from mice co‐stimulated with the same H1N1 influenza virus antigen showed enhanced differentiation of CD4+ T cells and secretion of important cytokines, such as IL‐6 and TNF‐α, compared with those in the control group, thus promoting Th1 cell differentiation, which was consistent with the results of humoral immunity. Furthermore, the BCG‐PSN adjuvant promoted an increase in central memory CD8+ cells and effector memory cells, which exert positive feedback on IgG production and humoral immunity.

Through virus challenge experiments, it was evident that BCG‐PSN could maintain mouse body weight at a stable level and significantly increased their survival rates, which might be attributed to the ability of BCG‐PSN adjuvanted vaccines to markedly elevate neutralizing antibody levels, reduce viral titers, and reduce levels of inflammatory factors. Additionally, during the safety analysis of BCG‐PSN, acute and long‐term toxicity observations were conducted based on previous studies [50]. Statistical analysis of weight data revealed no notable distinction between the group administered with the highest dose of BCG‐PSN and the PBS group. Moreover, no pathological phenomena were observed within 1 to 7 days post‐immunization and at days 14, 28, and 35 thereafter. As mentioned earlier, BCG‐PSN is frequently employed as an immunomodulator in clinical settings, with a well‐established safety record. In summary, the findings suggested that BCG‐PSN could markedly augment vaccine immunity and merits consideration as a prospective candidate for innovative immune adjuvants.

## 5 Conclusions

BCG‐PSN adjuvant exhibited favorable safety profiles and demonstrated an ability to elevate total antibody titers, particularly enhancing neutralizing antibody production when co‐administered with the H1N1 antigen. It increased cytokine levels and the proportion of CD8+ T lymphocytes, indicating its capacity to enhance cellular immunity. Upon viral challenge in mice, BCG‐PSN mitigated the production of inflammatory factors and reduced lung pathology, effectively protecting the mice. BCG‐PSN displayed heightened immunogenicity against a mixture of four antigens. BCG‐PSN is a reliable and efficient adjuvant for influenza vaccines, holding promise for enhancing vaccine efficacy and increasing immune responses.

## Author Contributions

**Sijing Yan:** conceptualization, methodology, software, data curation, formal analysis, writing – original draft. **Fan Yang:** methodology, investigation. **Jia Ji:** data curation, software. **Xiantian Lin:** data curation, methodology. **Ping Wang:** investigation, software. **Han Wu:** software, visualization. **Linfang Cheng:** formal analysis, validation. **Fumin Liu:** formal analysis, software. **Nanping Wu:** formal analysis, validation. **Hangping Yao:** resources, supervision, visualization. **Wade S.J. Wu:** conceptualization, software, validation, writing – review and editing. **Haibo Wu:** conceptualization, funding acquisition, project administration, resources, supervision, writing – review and editing.

## Conflicts of Interest

The authors declare no conflicts of interest.

## Data Availability

The datasets supporting the conclusions of this article are contained within the article and its supporting files.
